# Dermatomyositis associated with omalizumab therapy for severe asthma: a case report

**DOI:** 10.1186/s13223-019-0319-4

**Published:** 2019-01-17

**Authors:** Samira Jeimy, Pari Basharat, Fiona Lovegrove

**Affiliations:** 1 Division of Clinical Immunology and Allergy, Department of Medicine, Western University, London, ON Canada; 2 Division of Rheumatology, Department of Medicine, Western University, London, ON Canada; 3 Department of Medicine, Western University, London, ON Canada; 4 Division of Clinical Immunology and Allergy, St. Joseph’s Health Care London, Room B3-112, 268 Grosvenor Street, London, ON N6A 4V2 Canada

**Keywords:** Dermatomyositis, Omalizumab, Adverse drug reaction, Biologics

## Abstract

**Background:**

Omalizumab is a humanized monoclonal antibody widely used for treatment of persistent allergic asthma and antihistamine-refractory chronic urticaria. Immediate adverse events to omalizumab are well characterized. Delayed anaphylactoid and serum sickness-like reactions have also been described; however, their relationship to the drug remains uncertain, and the frequency is unknown.

**Case presentation:**

We present a case of a 59-year old female who developed amyopathic dermatomyositis (DM) after receiving omalizumab infusions for steroid-refractory severe asthma. After 6 months of omalizumab, the patient developed an erythematous, intensely pruritic cutaneous eruption. Skin biopsy indicated nonspecific features of dermatitis. However, neither topical corticosteroids nor gabapentin and maximal doses of multiple antihistamines gave her relief. On follow-up clinical exam 8 months later, she had classic cutaneous features of dermatomyositis, with confirmatory repeat skin biopsy. Laboratory investigations revealed negative myositis specific antibodies, positive antinuclear antibody, and negative anti-histone antibodies. Creatine kinase, lactate dehydrogenase, aspartate aminotransferase, alanine aminotransferase levels and C-reactive protein were also within normal limits. These findings supported the clinical impression of amyopathic DM. The patient’s symptoms improved with oral corticosteroid therapy. A malignancy screen was negative. There was no evidence of end organ dysfunction.

**Conclusions:**

Dermatomyositis is not a known adverse effect of omalizumab therapy. DM has a low incidence, but potentially life threatening consequences. Amyopathic DM may represent up to 21% of cases of DM, with similar risks of malignancy and end organ dysfunction. DM has been associated with biologic therapy. Using the Naranjo adverse drug reaction (ADR) probability scale, our patient had a “probable” omalizumab related ADR. A more likely explanation is that the patient had underlying DM that remained occult due to chronic corticosteroid therapy. Our case highlights the need for clinical vigilance and maintenance of a broad differential when patients on biologic therapies present with cutaneous eruptions. In our patient, the cutaneous clinical features of DM became pronounced over serial assessments. Laboratory markers may be deceptively normal, as in amyotrophic DM, or confounded by ongoing corticosteroid therapy. There are important clinical implications of prompt diagnosis, given the association of DM with end organ disease including interstitial lung disease, and possible concomitant malignancy.

## Background

Omalizumab is a recombinant humanized monoclonal antibody that selectively binds to human IgE. It has been used since 2003 for treatment of persistent allergic asthma and antihistamine-refractory chronic spontaneous urticaria. Immediate adverse events to omalizumab are well characterized, with anaphylaxis occurring in approximately 1 in 1000 patients. Delayed anaphylactoid and serum sickness-like reactions have also been described in case reports [1]; however, their relationship to the drug remains uncertain, and the frequency is unknown.

Dermatomyositis (DM) is not a known adverse effect of omalizumab therapy. DM is a chronic inflammatory myopathy with a low incidence (9.63 cases per 1 million) [2], but potentially life threatening consequences, with end-organ involvement of the respiratory [(e.g., interstitial lung disease (ILD)], cardiac, and gastrointestinal systems. Although most cases are idiopathic, the risk of malignancy is increased fivefold, and targeting of muscle auto-antigens by the tumour immune response is a hypothesized mechanism [2, 3]. A subset of dermatomyositis termed amyotrophic DM (historically called DM sine myositis) is a condition in which patients have characteristic skin manifestations without muscle weakness or abnormal muscle enzymes. Amyopathic DM may represent up to 21% of cases DM; however, up to 41% of patients with DM may be misclassified at their initial visit, particularly as up to 20% do not have detectable autoantibodies [4]. The rates of complications (e.g., ILD) and risk of malignancy are similar between individuals with amyopathic and classic DM [2, 3].

Dermatomyositis can be precipitated by drugs, most commonly hydroxyurea and statins [5]. DM has been associated with biologic therapy, including tumour necrosis factor (TNF) inhibitors [6], tyrosine kinase inhibitors [5] and the cytotoxic T-lymphocyte antigen 4 blockade agent ipilimumab [7]. The mechanism is not known, and establishing causation is challenging. In a literature review of 70 case reports of drug-induced DM on the MEDLINE database, published between 1950 and 2007, 60% of individuals had underlying, possibly predisposing autoimmune or malignant conditions [5]. The median timeframe between drug exposure and onset of DM was 2 years, ranging from 48 h after carticaine injection for tooth extraction to 11 years after penicillamine administration in a patient with rheumatoid arthritis. Muscular and pulmonary involvement was present in a minority of cases (39% and 11%, respectively). It remains unclear whether the drugs propagated development of an underlying disorder, functioned synergistically with predisposing factors, or initiated the disease.

In the next section, we describe the first reported case of dermatomyositis associated with omalizumab therapy.

## Case presentation

**Fig. 1:**
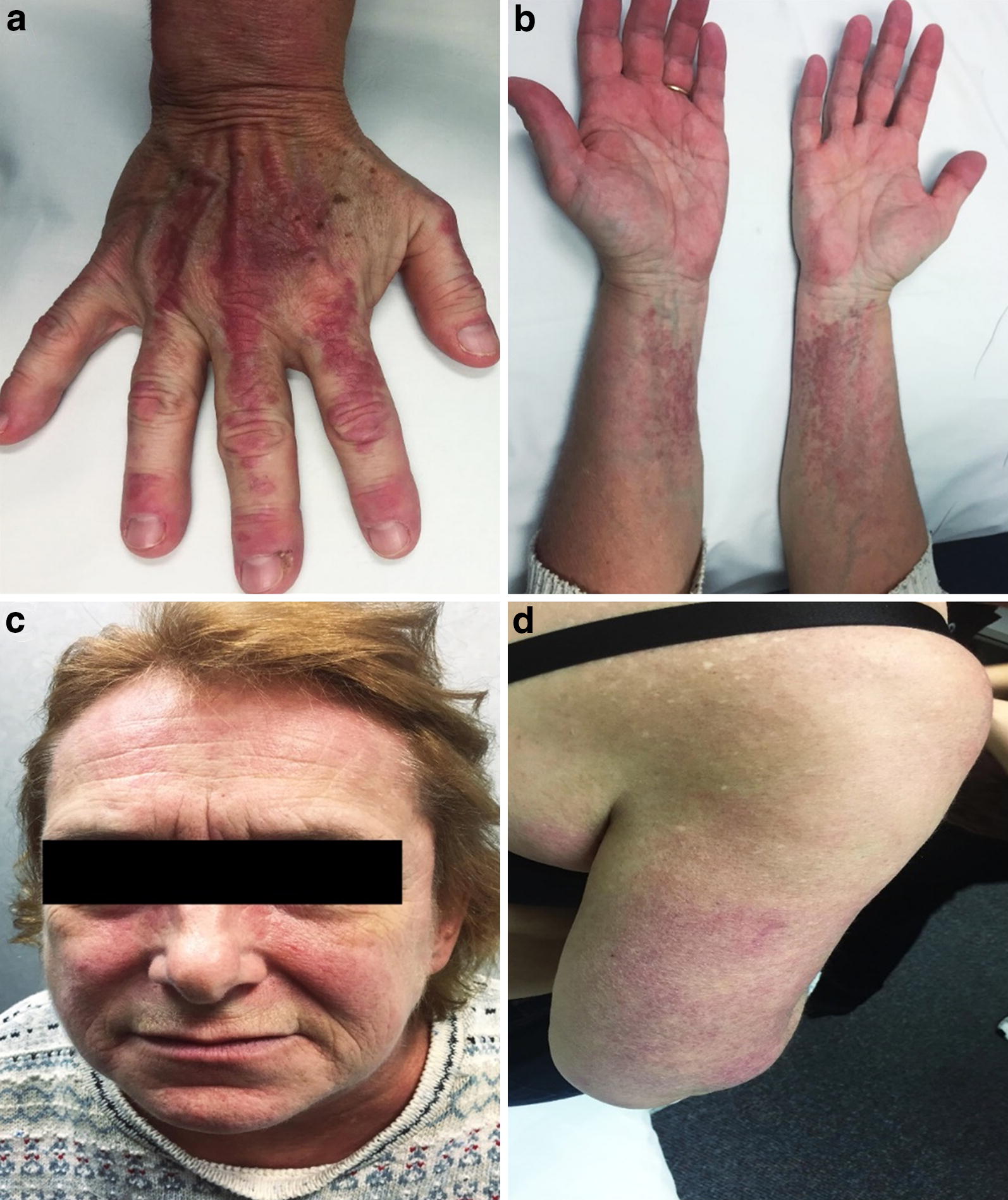
Clinical findings of dermatomyositis. The patient had erythematous papules over the dorsal knuckles (Gottron’s papules), with peri-ungual erythema and cuticle hypertrophy (**a**). Violaceous erythematous plaques were noted on the volar forearms (**b**) and posterior arms (Shawl sign; **d**). Also noted was photo distributed violaceous erythema of the forehead and midface, involving the nasolabial folds, as well as erythema and edema of the upper eyelids (heliotrope rash) (**c**). Poikiloderma with a mottled pattern of hyper-pigmented and hypo-pigmented macules interspersed with telangiectasia was noted on the upper back (**d**)

We present a case of a 59-year old female patient who developed amyopathic DM after receiving monthly omalizumab injections for severe asthma, followed by a Respirologist. Omalizumab had been prescribed as per current Canadian indications for severe, steroid-refractory asthma. The patient had no previous history of musculoskeletal or cutaneous problems, and her atopic history entailed asthma with dust mite sensitization. She had a 3-year history of chronic oral corticosteroid use, with greater than nine courses per year. After discontinuation of oral corticosteroids, followed by 6 months of omalizumab therapy, she developed an erythematous, intensely pruritic cutaneous eruption. For the next 3 months, with each subsequent dose of omalizumab, the eruption worsened, and omalizumab was discontinued. The patient presented to the Dermatology clinic and was noted to have violaceous erythema in a photo-exposed distribution. A skin biopsy was performed to rule-out DM or cutaneous lupus; however, only non-specific features of dermatitis were seen on histopathology. The patient started treatment with topical corticosteroids; however, neither this nor gabapentin and a combination of antihistamines (diphenhydramine, cetirizine, and hydroxyzine at maximal doses) gave her relief of the rash or intense pruritus. On follow-up clinical exam 8 months later, she had erythematous papules overlying the dorsal metacarpophalangeal joints, violaceous erythema affecting the face and upper eyelids, photo-distributed poikiloderma on the neck and shoulders, and scattered telangiectasia (Fig. 1). Hand exam indicated cuticular hypertrophy and peri-ungual erythema. There was symmetrical upper and lower extremity proximal muscle weakness (Medical Research Council grade 4+/5 at deltoids and hips).

Further laboratory investigations at the 8-month follow up visit included repeat skin biopsy and bloodwork for rheumatologic markers. A repeat skin biopsy showed interface dermatitis with focal thickening of the basement membrane. There was dermal lymphocytic infiltration and focal increase in dermal mucin. Laboratory investigations revealed a positive antinuclear antibody (titer > 1:640) with a speckled pattern. Screening by the extractable nuclear antigen (ENA) panel, which includes the myositis specific antibodies anti Ro/SSA, anti Jo-1, and anti-Scl70, was negative. Anti-histone antibodies were not detected. There was no eosinophilia. Creatine kinase, lactate dehydrogenase, aspartate aminotransferase, alanine aminotransferase levels and C-reactive protein were also within normal limits. Taken together, these findings supported the clinical impression of amyopathic DM.

The patient was referred to Rheumatology clinic and started on oral corticosteroid therapy (prednisone 1 mg/kg/day) (now 8 months after stopping omalizumab). Within days of starting prednisone, the patient’s eruption and pruritus improved dramatically. Prednisone was tapered slowly over 6 months, and methotrexate and hydroxychloroquine were started as steroid sparing agents. A malignancy screen including serial complete blood count and differential, computed tomography of the thorax, abdomen, and pelvis, Papanicolaou test, colonoscopy, and mammogram, was negative. There was no evidence of ILD, cardiac arrhythmia, conduction abnormalities, or pulmonary hypertension. Barium swallow was normal. As there was no objective evidence of muscle weakness on serial physical exams by a Rheumatologist, electromyography studies were not pursued. The patient is currently followed in Rheumatology and Respirology clinics. No new biologic agents have been added to her treatment regimen.

## Discussion and conclusions

Using the Naranjo adverse drug reaction (ADR) probability scale (Table 1), our patient had a “probable” omalizumab related ADR [8]. This score is driven by the temporal correlation of clinical symptoms after initial administration of omalizumab, followed by slow improvement after its discontinuation. An alternate explanation is that the patient had underlying DM that remained occult due to chronic corticosteroid therapy. This is supported by the clinical finding of persistent cutaneous symptoms 2 years after discontinuation of omalizumab therapy (the average serum elimination half-life of omalizumab is 26 days). Unmasking of autoimmune illnesses has been reported with omalizumab and eosinophilic granulomatosis with polyangiitis, previously believed to represent an ADR and now thought to relate to weaning of oral corticosteroids [9].

**Table 1:** The Naranjo scale for adverse drug reaction assessment. Adapted from [8]

	Question	Yes	No	Don’t know score
1.	Are there previous conclusive reports on this reaction?	+ 1	0	0
2.	Did the adverse event appear after the suspected drug was administered?	+ 2	− 1	0
3.	Did the adverse reaction improve when the drug was discontinued, or a specific antagonist was administered?	+ 1	0	0
4.	Did the adverse reaction reappear when the drug was readministered?	+ 2	− 1	0
5.	Are there alternative causes (other than the drug) that could on their own have caused the reaction?	− 1	+ 2	0
6.	Did the reaction reappear when a placebo was given?	− 1	+ 1	0
7.	Was the drug detected in the blood (or other fluids) in concentrations known to be toxic?	+ 1	0	0
8.	Was the reaction more severe when the dose was increased, or less severe when the dose was decreased?	+ 1	0	0
9.	Did the patient have a similar reaction to the same or similar drug in any previous exposure?	+ 1	0	0
10.	Was the adverse event confirmed by any objective evidence?	+ 1	0	0

Scores of ≥ 9, 5–8, 1–4, ≤ 0 indicate definite, probable, possible, and doubtful reactions, respectively

Total score

Likelihood that the adverse reaction was drug related

≥ 9 = highly probable

5–8 = probable

1–4 = possible

≤ 0 = doubtful

This case highlights the need for clinical vigilance and maintenance of a broad differential diagnosis when patients on biologic therapies present with cutaneous eruptions or muscle weakness. In our patient, the cutaneous clinical features of DM became pronounced over serial assessments, and she was initially treated for atopic dermatitis on the basis of clinical examination and skin biopsy. Laboratory markers may be deceptively normal, as in our case with amyotrophic DM, or confounded by ongoing corticosteroid therapy, which can be prescribed for nonspecific cutaneous eruptions. There are important clinical implications of prompt diagnosis, given the association of DM with end organ disease including ILD, and possible concomitant malignancy. Detailed reporting of adverse events in post-marketing surveillance of biologic therapy is important to raise awareness of uncommon but potentially life threatening presentations.

## Abbreviations

IgE: immunoglobulin E
DM: dermatomyositis
ILD: interstitial lung disease
TNF: tumour necrosis factor
ENA: extractable nuclear antigen
ADR: adverse drug reaction
